# Perspectives on Mechanisms Supporting Neuronal Polarity From Small Animals to Humans

**DOI:** 10.3389/fcell.2022.878142

**Published:** 2022-04-19

**Authors:** Carlos Wilson, Ana Lis Moyano, Alfredo Cáceres

**Affiliations:** Centro de Investigación en Medicina Traslacional Severo R. Amuchástegui (CIMETSA), Instituto Universitario de Ciencias Biomédicas de Córdoba (IUCBC), Córdoba, Argentina

**Keywords:** neurons, asymmetries, PARs, Rho GTPases, cytoskeleton, epigenetics, biophysics, human iPSCs

## Abstract

Axon-dendrite formation is a crucial milestone in the life history of neurons. During this process, historically referred as “the establishment of polarity,” newborn neurons undergo biochemical, morphological and functional transformations to generate the axonal and dendritic domains, which are the basis of neuronal wiring and connectivity. Since the implementation of primary cultures of rat hippocampal neurons by Gary Banker and Max Cowan in 1977, the community of neurobiologists has made significant achievements in decoding signals that trigger axo-dendritic specification. External and internal cues able to switch on/off signaling pathways controlling gene expression, protein stability, the assembly of the polarity complex (i.e., PAR3-PAR6-aPKC), cytoskeleton remodeling and vesicle trafficking contribute to shape the morphology of neurons. Currently, the culture of hippocampal neurons coexists with alternative model systems to study neuronal polarization in several species, from single-cell to whole-organisms. For instance, *in vivo* approaches using *C. elegans* and *D. melanogaster,* as well as *in situ* imaging in rodents, have refined our knowledge by incorporating new variables in the polarity equation, such as the influence of the tissue, glia-neuron interactions and three-dimensional development. Nowadays, we have the unique opportunity of studying neurons differentiated from human induced pluripotent stem cells (hiPSCs), and test hypotheses previously originated in small animals and propose new ones perhaps specific for humans. Thus, this article will attempt to review critical mechanisms controlling polarization compiled over decades, highlighting points to be considered in new experimental systems, such as hiPSC neurons and human brain organoids.

## The Role of Symmetrical and Asymmetrical Structures in Living Organisms

The physiological properties of living organisms depend on their three-dimensional conformation where symmetries and asymmetries play a crucial role. Different types of animals share an anatomical property named “bilateral symmetry,” reproducing tissues, organs and functions in both sides of the body. The central nervous system is particularly symmetric; the brain has two hemispheres narrowly interconnected to integrate and execute functions in response to inner and outer signals. Similarly, the spinal cord receives and projects afferent and efferent neurons from the left and right horns to innervate target tissues involved in perception and locomotion. Consequently, anatomical symmetries guarantee the proper coordination and integration of inputs to preserve homeostasis and survival.

Asymmetries are also instrumental for compartmentalization and functional specialization at various levels of organization, ranging from single cells to whole organisms. In this regard, brain asymmetry is a conserved characteristic of the vertebrate nervous system that contributes to improve its functioning (Levy, 1977). The habenula, a small brain nucleus connecting the basal forebrain with the midbrain, has served as a model system for studying signaling pathways leading to left-right asymmetries in the brain (Aizawa, 2013). On the other hand, inner regions of the human brain have asymmetries undetected in other mammals. For instance, language processing occurs in brain areas overdeveloped in the right hemisphere, whereas visuospatial information is mostly processed in the left hemisphere (Toga and Thompson, 2003). The presence of brain petalias—protrusions of one hemisphere into the other—are only seen in the human brain and their physiological relevance is still a matter of research (Gotts et al., 2013; Olulade et al., 2020). In fact, lateralization of cognitive functions is a very unique human condition, especially evident in the nervous system (Toga and Thompson, 2003). Therefore, asymmetries are fundamental to confer specialization for higher biological and cognitive tasks.

In this article we will discuss ancient molecular circuits to establish and maintain neuronal polarity, one of the most paradigmatic cases of programmed asymmetry in cells. Overall, the evidence collected in this article suggests that independently of animal complexity, molecular switches driving cell polarity and asymmetries are conserved across cell types, tissues and species.

### Polarization is a Paradigmatic Case of Asymmetry

The concept *polarization* is commonly used in biology to highlight asymmetries at various levels, ranging from biological molecules to tissues. For example, microtubules (MT) are intrinsically polarized polymers, exhibiting fast (+) and slow (-) growing ends in their extremes (Mitchison and Kirschner, 1984; Kirschner and Mitchison, 1986). At the cellular level, neurons are paradigmatic cases of polarization and we often call them “polarized cells.” Nevertheless, it is important to highlight that symmetrical cells do not exist and this concept is used to reinforce the absence of macroscopic asymmetries. Accordingly, intrinsic and extrinsic signals, such as environmental cues and cell-cell/cell-matrix interactions, will polarize any cell type in different degrees. Therefore, it is important to clearly state that cells are intrinsically asymmetric.

At the tissue level, epithelia have been extensively studied because of their characteristic apicobasal and basolateral polarities, determined by tight and adherent junctions as well as extracellular cues (McCaffrey and Macara, 2012). During central nervous system (CNS) development, embryonic gradients of morphogens shape the neural tube (neuroepithelium) into the anterior-posterior (AP) and dorsal-ventral (DV) axis, creating the coordinates to position the brain (anterior) and the spinal cord (posterior). Therefore, tissue morphogenesis is driven by chemical gradients to promote asymmetries, polarization and functional specialization.

The asymmetries of living organisms have been the focus of attention for decades, especially during embryonic development. How does the embryo diversify into multiple cell types from a single-cell zygote? Although this question still is under research, at present we know that asymmetric divisions of blastomeres are critical. During the 1980s several articles reported what we currently know as *Partitioning-defective (Par)* genes, a family of genetic determinants able to polarize the zygote (and any cell type) into the AP axis. Consequently, daughter cells (blastocysts) receive cytosolic constituents unequally, diversifying cellular lineages in the offspring.

### Neuronal Polarization

The neuron is a dramatic case of cellular polarization, manifested by the axonal and dendritic compartments that emerge during embryonic brain development. It is important to note that there are dozens of neuronal phenotypes and some of them display higher levels of polarization than others. In this regard, hippocampal/cortical pyramidal neurons and cerebellar Purkinje cells are two of the most polarized neuronal types of the mammalian brain. Interneurons, mesencephalic neurons, as well spinal motor neurons and dorsal root ganglion cells (DRGs) are also asymmetric cells, although with simpler forms and their *polarity* has not been studied as deep as in pyramidal neurons.

Neuronal polarization is not just a morphological feature. In fact, it is a biochemical compartmentalization determining the transmission of electrochemical currents along the neuron and consequently their function (Arimura and Kaibuchi, 2007; Hedstrom et al., 2008; Cáceres et al., 2012; Huang and Rasband, 2018). Whilst dendrites are highly specialized in receiving inputs, usually encoded by neurotransmitters and/or mechanical stimuli, the axon propagates the action potential anterogradely (from the Soma to the axonal tip) releasing vesicles containing peptides and neurotransmitters into the synaptic cleft. Thus, neuronal polarization involves morphological, biochemical and physiological transformations supporting the wiring and connectivity of the nervous system.

## Lessons From Rodents: The Axon-Dendrite Polarity

Most of our knowledge regarding the development and physiology of neurons comes from observations made in experimental animal models, including invertebrates such as worms (*C. elegans*), the fruit fly (*D. melanogaster*) and small mammals like rats (*R. norvegicus*) and mice (*M. musculus*) (Kaech and Banker, 2006; Rolls and Jegla, 2015; Harterink et al., 2016; Wilson et al., 2020b; He et al., 2020). In the last decade, the reprogramming of skin fibroblasts into *induced pluripotent stem cells* (iPSCs) has opened the possibility of studying human neuronal polarization *in vitro* either culturing single-neurons or brain organoids (Takahashi and Yamanaka, 2006; Takahashi et al., 2007; Lancaster et al., 2013). Although we are far from reproducing human *in situ* environment precisely, culturing human iPSC-derived (hiPSC) neurons is a substantial advance in the field.

**FIGURE 1:**
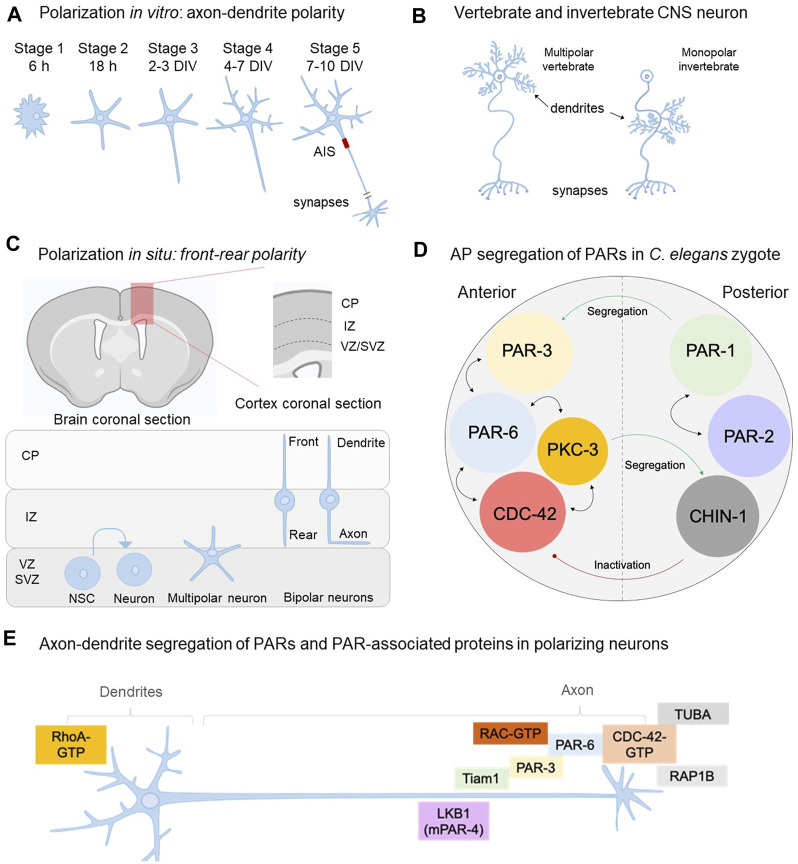
PARs, the polarity complex and their role on neuronal polarity acquisition. **(A)** Polarization of neurons *in vitro*—isolated from embryonic murine hippocampi or brain cortices—occurs through the sequential transformation of rounded postmitotic cells (*stage1*) to fully polarized neurons able to form synapses with other neuronal and glial cells (*stage 5*). **(B)** Representative multipolar vertebrate motor neuron with dendritic process at the soma and monopolar invertebrate motor neuron where dendritic branches develop from a thin primary neurite that extends towards the neuropil. **(C)** Polarization of cortical neurons *in situ* starts after neurogenesis in the VZ (ventricular zone) of the developing brain cortex enriched in neural stem cells (NSC). Then, postmitotic neurons adopt a multipolar phenotype and migrate from the SVZ (subventricular zone) to the IZ (intermediate zone) adopting the front-rear polarity. Dendrites and the axon will emerge from front (leading) and rear (trailing) processes, respectively. **(D)** Segregation of PARs in the *C. elegans* zygote establish a feedforward circuit aiming the maintenance of the AP axis. This circuit is reproduced across species, cell types and developmental stages to allow cell polarity. For example, PAR-1 phosphorylates PAR-3; a modification that allows PAR-3 anterior segregation. In addition, CHIN-1 is a CDC-42 GTPase located in the posterior domain, preventing CDC-42 activation outside the anterior pole. Thus, PARs establish mutual exclusion associations to maintain the circuitry. Bidirectional arrows mean interaction/recruitment. **(E)** Distribution of PARs in polarizing neurons (stage 2–3 transition and stage 3 *in vitro*) allows axon-dendrite formation in polarizing neurons *in vitro*. PAR-3, PAR-6, PKC-3, CDC-42 (namely “polarity complex, PC”) assemble with Tiam1 (Rac’s GEF) and RAC through PAR-3 and PAR-6. RAP1B (CDC-42 activator) and TUBA (CDC-42’s putative GEF) also segregate into the nascent axon supporting CDC-42 activation (GTP state) and consequently the PC.

The culture of hippocampal neurons isolated from embryonic rat brains by Gary Banker and Max Cowan in 1977 was a breakthrough for modern neurobiology in several aspects (Banker and Cowan, 1977). This model system allowed the neuroscience community to study CNS neurons at the single cell level distinguishing subcellular domains and compartments difficult to visualize using brain and nerve tissues (Bartlett W. P. and Banker G., 1984a, Bartlett W. and Banker, G. 1984b; Caceres et al., 1986; Banker, 2018). Moreover, cultured hippocampal neurons revealed an intrinsic program of morphological transformations defining the axon and dendrites, a process referred as *the establishment of neuronal polarity* (Dotti et al., 1988; Banker, 2018). In culture, neurons isolated from the rat embryonic hippocampus (E18) undergo progressive transformations until reaching a highly polarized morphology (Figure 1A). Initially described as a 5-stage process, the acquisition of neuronal polarity must achieve several milestones (Dotti et al., 1988). First, postmitotic neurons plated on plastic or glass dishes (precoated with an inert agent such as poly-L-lysine, PLL) are round cells surrounded by an actin-rich structure (lamella) (*stage 1*; first 6 h in culture). Then, round neurons experience plasma membrane deformations leading to neurite sprouting, reaching a multipolar conformation named *stage 2*. Neurite outgrowth is driven by actin microfilaments (FA) and MT dynamics (Caceres et al., 1984; Caceres and Kosik, 1990; Bradke and Dotti, 1999; Conde and Cáceres, 2009; Stiess and Bradke, 2011). At this stage, neurons are considered symmetric since neurites are morphologically equivalent. However, as we will discuss in the following sections, external and internal signals select one of the neurites pushing its growth considerably, taking the lead to become the axon (*stage 3*; 36–48 h in culture). Far from being a linear transformation, the axon-like neurite is not completely committed yet and minor neurites still have time to be re-specified into the future axon. Accordingly, the extension of the nascent axon occurs through growth and retraction phases (Dotti et al., 1988), resembling the dynamic instability of MT; in fact, both MT and FA are the driving force of neuronal polarization. A seminal work by Bradke and Dotti (1999) described that local dismantling of FA in the growth cone of a minor neurite with cytochalasin D is enough to induce its growth as the axon. By this time, mitochondria, lysosomes and trafficking vesicles move into the prospective axon, supporting the delivery of cellular constituents needed for axonal extension (Bradke and Dotti, 1997). Overall, axonal specification is a dynamic process that takes time and meanwhile, any minor neurite can take the lead under proper growth conditions. Accordingly, during stage 2–3 transition the cellular symmetry is broken and thus it is considered the most critical stage of polarization (Cáceres et al., 2012).

Once the axonal domain is defined, minor neurites develop into future dendrites, experiencing branching and growth (*stage 4*, 4–7 days in culture). Time-lapse video-microscopy of hypothalamic or amygdala neurons in culture indicates that these cells undergo a similar sequence of morphological transformations to generate a single axon and multiple dendrites (Díaz et al., 1992; Lorenzo et al., 1992).

Aside from this, it should be kept in mind that at stage 4, cultured hippocampal neurons are still electrically immature. Embryonic neurons show notorious differences with mature stages on handling ion diffusion required for neurotransmission. For instance, embryonic expression of the Na^+^-K^+^-Cl^-^ symporter elevates intracellular levels of chloride (Cl^−^), being gamma aminobutyric acid (GABA) the principal excitatory signal in immature neurons (Mir et al., 2020; Lombardi et al., 2021). In this regard, voltage-dependent ion channels and synaptic proteins are expressed once the axon is specified. In fact, Nav and Kv channels triggering the action potential are recruited to the *axon initial segment* (AIS), an intra-axonal domain assembled at 7–10 days in culture (*stage 5*) or after birth *in situ* (Galiano et al., 2012; Huang and Rasband, 2018; Leterrier, 2018). Voltage-dependent ion channels are recruited by ankyrins (AIS proteins), polarizing the flux of the electrical activity (Hedstrom et al., 2008; Huang and Rasband, 2018). Moreover, the AIS serves as a physical barrier avoiding free diffusion of dendritic constituents into the axon. Consequently, dismantling the AIS leads to the entry of dendritic components that finally respecify the axon in dendrites (Hedstrom et al., 2008; Galiano et al., 2012). In contrast, neurons displaying multiple axons, revealed by Tau-1 staining and expression of presynaptic markers such as vGLUT1 and synaptophysin, also show multiple AIS (Muñoz-Llancao et al., 2015). However, the electrical conductance of these processes remains to be tested. Finally, both axons and dendrites express the synaptic machinery required for neurotransmission and there are many common features but also fundamental differences between vertebrate and invertebrate neurons (Smarandache-Wellmann, 2016) (Figure 1B). Thus, polarization has deep consequences for the wiring and connectivity of the nervous system.

Other model systems, such as cerebellar macroneurons (Caceres et al., 1992), sympathetic neurons (Higgins et al., 1988) or retinal photoreceptors (Adler, 1986) (Adler and Madreperla, 1990) have also been used to study neuronal polarization in culture; unfortunately, whether or not these cells acquire a full mature polarized phenotype has remained unexplored. The case of sympathetic neurons is particularly interesting since dendrite formation is highly dependent on bone morphogenetic factor 7 (BMP7), a member of the transforming growth factor β (TGF β) - superfamily; in the absence of this factor these neurons only elaborate the axon (Lein et al., 1995). Importantly, this study was one of the first showing that neuronal shape and polarization can be regulated by specific signals (Higgins et al., 1997).

### Similarities and Differences Between *In Vitro* and *In Situ* Polarization

Polarization has also been studied during the development of the embryonic cerebral cortex *in situ*, showing similarities and differences. After neurogenesis, cortical neurons experience migration and polarization almost simultaneously, moving from the ventricular zone (VZ) to the cortical surface (Kriegstein and Noctor, 2004; Barnes and Polleux, 2009; Namba et al., 2014; Xu et al., 2015) (Figure 1C). During neurogenesis, the apical radial glia (aRG) located in the VZ divides and differentiates into basal precursors (BP), which differentiate into neural stem cells (NSC) and neurons. Consequently, BPs, NSC and neurons migrate through cellular interactions with the radial glial cell (RGC) to inhabit the upper layer of the cortex supporting corticogenesis (Kalebic and Namba, 2021). Accordingly, a major difference of *in situ* polarization is that it occurs simultaneously with neurogenesis and migration, involving neuron-glia and tissue interactions un-experienced by isolated neurons in culture.

Immediately after neurogenesis, newborn neurons sprout neurites adopting a multipolar phenotype similar to stage 2 *in vitro* (Kriegstein and Noctor, 2004; Kalebic and Namba, 2021). Then, multipolar neurons develop a front (dendrite) and a rear (axon) process, clearly evident in the interphase between the SVZ and the IZ (Kriegstein and Noctor, 2004; Namba et al., 2014; Kalebic and Namba, 2021) (Figure 1C). The front-rear polarity—also named bipolar morphology—is characteristic of *in situ* polarization and represents a major difference with *in vitro* observations (Figures 1A,C). Cellular contacts between the front and rear process with the RGC allows radial migration of neurons, working as a track shaping the bipolar morphology (Xu et al., 2015). In contrast, cultured neurons grow in a bidimensional matrix lacking RGC interactions. Moreover, *in vitro* polarization begins with postmitotic neurons, whilst *in situ* it involves asymmetrical divisions of NSC and neurogenesis leading newborn neurons (Kriegstein and Noctor, 2004). Collectively, these examples reinforce the importance of the environment in shaping neurons beyond their intrinsic polarizing program; in this regard, N- cadherin, a cell adhesion molecule, and Reelin, a large extracellular matrix molecule, play pivotal roles in regulating cortical neuronal migration and polarization (Gärtner et al., 2015; Santana and Marzolo, 2017).

The neuronal lineage derives from the embryonic neuroepithelium, which in turn emerges when the neural tube is developed (Govek et al., 2011). This tissue holds epithelial properties such as the apicobasal polarity and extends to the plasma membrane with different protein and lipid compositions that seems to determine the establishment of neuronal polarity (Bonifacino, 2014). For example, neurogenesis starts in the VZ of the brain cortex, which anatomically corresponds to the apical zone of the telencephalon; the anterior portion of the primitive embryonic brain (Govek et al., 2011). Thus, the apicobasal polarity observed in epithelia is reproduced along brain development and particularly the brain cortex. In this regard, front-rear polarity resembles apicobasal polarity, showing a leading neurite (front; dendrite) and a trailing process (rear; axon). It seems that developing neurons recapitulate polarizing events of the embryo through a sort of “polarity memory.” On this matter, a recent article discusses about the inheritance of cell polarity and its role on brain development (Kalebic and Namba, 2021). Authors hypothesize that polarization of neurons has an inheritable component relaying on the asymmetric divisions of aRG and BP in the brain cortex. Moreover, enlargement and complexity of the human brain cortex could be the result—at least in part—of additional mitotic cycles experienced by BPs before neurogenesis and glial differentiation (Kalebic and Namba, 2021).

## Intrinsic Programs Supporting Polarization

Although *in vitro* and *in situ* polarization have differences (i.e.: axon-dendrite vs. front-rear polarity, respectively), it is evident that neurons hold a cell-autonomous program of differentiation. The repertoire of polarizing molecules is vast, including trophic factors, neurotransmitters, hormones, peptides, lipids, nucleotides, ions and more. Thus, polarity acquisition is the result of a dynamic balance between positive (*propolarizing*) and negative (*antipolarizing*) external and internal signals (Takano et al., 2019). Accordingly, polarization is a step-by step rather than a linear transformation, and neurons can redefine polarity in early stages. Most of the external ligands are decoded by plasma membrane receptors regulating molecular circuits to allow polarization. Particularly, the polarity complex is an ancient and evolutionary conserved group of genes/proteins aiming the development and maintenance of cellular asymmetries, controlling vital processes in all metazoans, from embryo development to neuronal polarization.

### The Polarity Complex is a Genetically-Encoded Machinery Driving Asymmetries

The polarity complex (PC) was described in *C. elegans* by Kenneth Kemphues, James Priess, Diane Morton and Niansheng Cheng in 1988 when studying embryo and tissue development using the worm as a model system (Kemphues et al., 1988) (Figure 1D and Table 1). By introducing deleterious mutations in a large set of genes, they found a subset of mutants unable to partition blastocysts during the first mitosis of the zygote; collectively, these genes were called *Partitioning-defective* (*Par* genes). *Par* genes encode a molecular circuit promoting polarity in virtually all cells and tissues, aiming the formation of asymmetries at different levels, ranging from embryogenesis to neuronal polarization (Böhm et al., 1997; Brazil and Hemmings, 2000; Kemphues, 2000; Jan and Jan, 2001; Shi et al., 2003; Brajenovic et al., 2004).

**TABLE 1:** *Par* genes and PAR proteins in *C. elegans* and mammals.

Protein	*C. elegans* (description)	*Mammals*	Zygote*	Neuron
PAR-1	Ser/Thr K	MARKs	pPAR	Unknown
PAR-3	Scaffold	mPAR-3	aPAR	Enriched axon—Soma Shi et al. (2003)
PAR-4	Ser/Thr K	LKB1	equivalent	Axon
PAR-5	14-3-3 protein	14-3-3 proteins	equivalent	Unknown
PAR-6	Scaffold	mPAR-6	aPAR	Enriched axon—Soma Shi et al. (2003)
PKC-3	Ser/Thr K	aPKC-3	aPAR	Unknown
CDC-42	Small GTPase	CDC42	aPAR	Enriched axon—Soma Schwamborn and Püschel, (2004)
CHIN-1	GAP	Unknown	pPAR	Unknown
LGL-1	Tumor suppressor	Unknown	pPAR	Unknown

Summarizes name and localization of PARs, in the worm and mammals according to the references discussed in the main text. * (McCaffrey and Macara, 2012; Lang and Munro, 2017).

### Overview of PAR Proteins in *C. elegans*

There are 10 PAR proteins in *C. elegans*, namely PAR-1 to PAR-6, PKC-3, CDC-42, CHIN-1 (CHImaeriN [RAC-GTPase-activating protein] homolog), and LGL-1 (*lethal giant larvae*) (Etemad-Moghadam et al., 1995; Tabuse et al., 1998; Gotta et al., 2001; Beatty et al., 2010; Hoege et al., 2010; Kumfer et al., 2010; Lang and Munro, 2017). Excluding PAR-2, only expressed in worms, remaining PARs are relatively conserved in all metazoans. PAR-1 and PAR-4 are Ser/Thr kinases (Guo and Kemphues, 1995; Watts et al., 2000), whereas PAR-2 is a RING finger protein stabilizing cell polarity (Boyd et al., 1996; Hao et al., 2006; Beatty et al., 2010; Lang and Munro, 2017). Of note, RING finger domains mediate ubiquitin transfer and consequently protein stability (Joazeiro and Weissman, 2000). In addition, PAR-3, PAR-5 and PAR-6 modulate protein-protein and membrane-protein interactions (Hung and Kemphues, 1999). For instance, PAR-3 holds three PDZ domains and works as a scaffold protein recruiting PAR-6 (Hung and Kemphues, 1999). Moreover, PAR-5 contains 14-3-3 domains and binds to a large variety of proteins such as PAR-1, supporting the establishment of the AP axis (Benton et al., 2002; Morton et al., 2002). Finally, PAR-6 is an adaptor protein, holding PDZ and CRIB domains (CDC-42/Rac interacting binding domains) interacting with PAR-3 and CDC-42 (Hung and Kemphues, 1999; Aceto et al., 2006).

Another member of PARs is CDC-42, a GTPase belonging to the subfamily of small Rho GTPases together with Rac and RhoA, among others (Hall, 1990, 1998; Ridley and Hall, 1992). Pioneering work by Allan Hall and collaborators established that Rho proteins modulate FA dynamics, with Rac and CDC-42 promoting lamellipodia and filopodia formation, respectively, and RhoA actin stabilization and stress fiber formation (Hall, 1990, 1998). Since then, Rho GTPases have been implicated in a wide variety of functions including a crucial role in the establishment of neuronal polarity exerting local control of FA and MT dynamics required for axonal formation and navigation (Gonzalez-Billault et al., 2012).

In addition, the GTPase activating protein (GAP) CHIN-1 also belongs to PARs and balances CDC-42 and Rac activities (Kumfer et al., 2010). Finally, the family is completed with the Ser/Thr kinase PKC-3 and the tumor suppressor protein LGL-1 (Tabuse et al., 1998; Hoege et al., 2010).

### The Interdependence of PARs, MT and FA to Establish the AP Axis

Immediately after fecundation PARs are uniformly distributed in the zygote together with RhoA and Myosin II. However, PAR-3, PAR-6, CDC-42 and PKC-3 segregate to the anterior zone (aPARs, for *anterior*) by mechanisms not clearly identified yet. In contrast, PAR-1, PAR-2, CHIN-1 and LGL-1 remain in the posterior domain (pPARs, for *posterior*) (McCaffrey and Macara, 2012; Lang and Munro, 2017). Several lines of evidence suggest that the site of sperm entry into the oocyte defines the posterior pole of the zygote (Goldstein and Hird, 1996; O’Connell et al., 2000; Sadler and Shakes, 2000). Accordingly, the sperm would supply the centrioles that assemble with the pericentriolar material of the oocyte to constitute the centrosome, defining the posterior domain and suggesting a role for MT (Cuenca et al., 2003; Bienkowska and Cowan, 2012). In addition, sperm-derived inhibitory cues (localized in the posterior domain) would reduce RhoA activity leading to actomyosin cortical flows supporting AP segregation (Munro et al., 2004; Motegi and Sugimoto, 2006). Therefore, the site of fecundation has a positional value defining the organization center for MT as well as early FA dynamics promoting the AP axis.

### Anterior and Posterior PAR Proteins Establish a Feed-Forward Circuit

PAR-2 recruits PAR-1 to the posterior domain of the zygote. In turn, PAR-1 phosphorylates PAR-3 avoiding its self-oligomerization and hence promoting its anterior segregation (Figure 1D). In addition, active CDC-42 (GTP bound) also localizes in the anterior domain and its activity seems to be downregulated by the GAP CHIN-1, mostly localized in the posterior pole, a distribution narrowly dependent on PKC-3. In this regard, the anterior localization of PKC-3 is achieved by the binding to PAR-3, PAR-6 and CDC-42. Finally, PAR-4 and PAR-5 are uniformly distributed in the zygote and their suppression disrupts the AP axis, affecting asymmetric cell divisions and polarization of the zygote (McCaffrey and Macara, 2012; Lang and Munro, 2017).

PARs are expressed in all metazoans (Table 1). For example, the Baz gene in *D. melanogaster* encodes Bazooka; the fly homologue of PAR-3 that assembles with PAR-6 and aPKC (Brazil and Hemmings, 2000; Jan and Jan 2001). The mammalian PAR-3 (mPAR-3) was firstly named *aPKC specific interacting protein* (ASIP) (Izumi et al., 1998; Brazil and Hemmings, 2000). As in flies, mPAR-3 distributes in the anterior pole of dividing stem cells, neuroblasts and sensory organ precursor (SOP) cells. PAR-3 orthologues share three conserved regions (CR1-3); CR2 holds PDZ domains for PAR-6 and aPKC recruitment (Izumi et al., 1998; Lang and Munro, 2017), whereas CR3 allows PAR-3/PAR-6/aPKC assembly (Soriano et al., 2016). Accordingly, CR3 phosphorylation by aPKC disassembles PAR-6 and aPKC (Ohno, 2001).

Overall, PARs establish a molecular circuit aiming the formation of asymmetries and cellular polarity (Figure 1D). As expected, they also play a crucial role in the establishment of neuronal polarity by controlling axon-dendrite formation. In the following sections we will analyze the circuitry established by PARs and their molecular partners to decode signals driving axon formation.

### The Polarity Complex Supports Neuronal Polarization and Axon Formation

In 2003, Shi and colleagues reported the expression and axonal segregation of PAR-3 and PAR-6 in cultured hippocampal neurons isolated from embryonic rat brains (Shi et al., 2003). This was the first of a series of articles published in the 2000s reporting the contribution of the PC to axon-dendrite formation. Accordingly, authors reported that PAR-3 and PAR-6 are enriched in the tip of the growing axon in stage 2–3 neurons (Figure 1E), although they are also detectable in the cell body to a lesser extent. *In situ*, the axon of polarizing cortical neurons grows towards the VZ, the anterior domain of the embryonic neuroepithelium (Govek et al., 2011). Thus, axonal enrichment of PAR-3 and PAR-6 resembles the anterior segregation observed in *C. elegans* zygote. However, distribution of PARs has not been assessed in the murine brain *in situ*.

The atypical protein kinase C (aPKC; PKC-3 in *C. elegans*) is a principal component for the polarization of hippocampal neurons in culture. Accordingly, pharmacological blockade of aPKC arrest neurons in a multipolar phenotype (stage 2), restraining axonal growth (Shi et al., 2003). However, distribution of aPKC in polarizing neurons remains to be evaluated. In *C. elegans*, PKC-3 phosphorylates the CR3 domain of PAR-3, supporting anterior segregation of PAR-3 and PAR-6 (Izumi et al., 1998; McCaffrey and Macara, 2012; Lang and Munro, 2017). In culture, stage 3 neurons show an axonal enrichment of phosho-aPKC (Thr 403/410), although native aPKC distribution remains to be tested (Shi et al., 2003). Overall, this data suggests that active aPKC, PAR-3 and PAR-6 segregate into the nascent axon of polarizing neurons.

### The Cross-Talk of Rho GTPases and the Polarity Complex

CDC-42, Rac and RhoA belong to the Rho family of small GTPases, molecular switches cycling between inactive (GDP-bound) and active (GTP-bound) states controlling cytoskeletal dynamics, cell contractility, migration and polarity (Hall, 1990, 1998; Ridley and Hall, 1992; Gonzalez-Billault et al., 2012). Their activities depend on the exchange of GDP by GTP catalyzed by guanine exchange factors (GEFs) proteins. Although Rho proteins can hydrolyze GTP into GDP to return to their basal state, this is enhanced by GTPase activating proteins (GAPs). Currently, there is a global consensus on the role of Rho proteins in the establishment of neuronal polarity (Gonzalez-Billault et al., 2012). In general terms, both Rac1 and CDC-42 favors polarization and axon formation by promoting FA dynamics required for axon extension and guidance. In fact, local instability of actin in the growth cones of developing neurites allows its specification and elongation as an axon (Bradke and Dotti, 1999). By contrast, RhoA counteracts their activities by promoting FA stabilization through the activation of downstream effectors like ROCK and CRMP-2 (Sayas et al., 2002; Conde et al., 2010; Xu et al., 2015; Dupraz et al., 2019; Wilson et al., 2020a). GSK3β, which is also activated by RhoA, is a major regulator of neurogenesis, polarization and cytoskeletal organization acting during asymmetric cell division, neuronal migration, polarization and axon extension. Several excellent reviews have addressed in detail the role of GSK3 signaling in neuronal development (Li, 2005; Hur and Zhou, 2010; Seira and Del Río, 2014).

Activation of CDC-42 and Rac1 is achieved by two simultaneous mechanisms. On one hand, they are recruited in the axon by the CRIB domain of PAR-6 (Brazil and Hemmings, 2000). On the other hand, PAR-3 binds to the Rac-specific GEF Tiam1, leading to Rac1 activation (Nishimura et al., 2005). As PAR-3 and PAR-6 establish a complex, Rac1 meets Tiam1 promoting polarization and axonal growth (Kunda et al., 2001; Kawauchi et al., 2003; Nishimura et al., 2005). Hence, Rac1 activity in polarizing neurons depends on the assembly of the PC.

Whilst worm CDC-42 localizes in the anterior domain of the zygote, cultured rat neurons show a preferential distribution in the nascent axon and growth cone of stage 2–3 neurons. As it occurs with aPKC, the activity of CDC-42 is locally regulated. In fact, the small GTPase Rap1b, an upstream activator of CDC-42 is enriched in the axonal tip of stage 3 neurons, suggesting a local regulation (Schwamborn and Püschel, 2004). Along this line of evidence, a recent article reported that the dynamin-binding protein (DNMBP) TUBA segregates into the axon working as GEF for CDC-42 (Urrutia et al., 2021). Collectively, these observations suggest local regulations favoring CDC-42 activation in axons.

Reinforcing this concept, the GTPase Rac1 is uniformly distributed in minor neurites of stage 2 cultured neurons. However, the Rac1-specific GEF Tiam1 shows a preferential distribution into the nascent axon and the axonal growth cone, exerting a local control of Rac1 (Kunda et al., 2001). By contrast, stage 2 and early stage 3 neurons display a polarized activity of the GTPase RhoA, being highly active in minor neurites (and their growth cones) and almost inactive along the growing axon (Conde et al., 2010; Wilson et al., 2020a) (Figure 1E). Hence, this data supports the notion that small GTPases provide positive and negative signals balancing cytoskeletal dynamics required for the polarization of central neurons (Conde and Cáceres, 2009; Cáceres et al., 2012; Wilson et al., 2020a). Rac1 and Tiam1 are important regulators of the neuronal PC and although they were not initially considered as part of the central core (as PAR proteins do), current evidence supports their inclusion as members of the neuronal PC.

## The Posterior PAR Proteins in Polarizing Neurons

### MARK2, Mammal Orthologue of PAR-1, Impairs Neuronal Polarization

Pyramidal rat neurons express orthologues of the worm pPARs. For instance, the microtubule affinity regulating kinase 2 (MARK2), human orthologue of PAR-1 (Böhm et al., 1997), determines the acquisition of polarity (Chen et al., 2006). MARK2 is a protein kinase targeting Doublecortin (DCX), MAP1B, MAP2 and Tau; all of them microtubule-associated proteins (MAPs) conferring MT stability required for axon extension (Conde and Cáceres, 2009). Consequently, phosphorylation of MAPs by MARK2 reduces their MT affinity, leading to instability and catastrophic events ultimately inhibiting axonal growth. Moreover, this phenotype is rescued when aPKC phosphorylates MARK2 at Thr 595 (Suzuki et al., 2004). In neurons, either MARK2 deletion or kinase-dead expression lead to multiple axon formation, displaying two or more Tau-1 positive neurites (Chen et al., 2006), a dephosphorylated epitope of Tau enriched in the distal axon (Mandell and Banker, 1996). Accordingly, Tau-1 levels increase after MARK2 suppression, suggesting a negative cross-talk between aPKC and MARK2. Along this line of evidence, overexpression of MARK2 increases Tau phosphorylation at Ser 262, reducing Tau-1 detection in the prospective axons and blocking the acquisition of polarity; a phenotype that is only rescued by the simultaneous expression of aPKC, PAR-3 and PAR-6 (Chen et al., 2006). Thus, neurons express both anterior and posterior PARs establishing a fluid crosstalk needed to break the symmetry of developing neurons.

### LKB1, Mammal Orthologue of PAR-4, Favors Axon Formation

Rat neurons express the Ser/Thr kinase LKB1 (also STK11), a mammal orthologue of the worm PAR-4 (Watts et al., 2000; Benkemoun et al., 2014) involved in the establishment of epithelial polarity of *D. melanogaster* and *X. laevis* (Martin and Johnston, 2003; Ossipova et al., 2003).

LKB1 establishes a complex with STRAD and MO25 to promote axon initiation and radial migration of rat cortical neurons (Barnes et al., 2007; Shelly et al., 2007). Suppressing LKB1 in cultured neurons prevents axon specification and extension, whilst overexpression drives to multiaxonic neurons. *In situ*, genetic suppression of LKB1 restrain axon formation, severely affecting cortical migration. In particular, genetic ablation of LKB1 shows a clear reduction of callosal and corticofugal axons, reinforcing the concept that LKB1 is a *propolarizing* protein (Barnes et al., 2007).

In culture, LKB1 shows a selective accumulation in one of the neurites of stage 2 neurons (12 h *in vitro*), as well as in axons of stage 3 (48 h *in vitro*) (Shelly et al., 2007) (Figure 1E). In this regard, the *protein kinase dependent of cyclic AMP* (PKA) phosphorylates LKB1 at Ser 431 enhancing its stability and promoting axonal segregation (Barnes et al., 2007). Consequently, phospho-LKB1 targets downstream effectors such as the polarity proteins SAD-A/B kinases, AMPK and AMPK-related kinases, such MARKs 1-4 (Lizcano et al., 2004; Barnes et al., 2007). LKB1 phosphorylates the T-loop Ser residue present in MARK2/3, which is conserved in all AMPK and AMPK-like proteins, including MARKs.

## Decoding Extrinsic Signals: The Role of TRK Receptors, PI3K and PIP3

Extracellular signals feed the circuitry of the polarity complex. There is a wide consensus on the classification of these factors into positive and negative signals, promoting or restraining polarization. For instance, BDNF, insulin, IGF-1 and TFG-ß, among others, bind to their plasma membrane receptors triggering activation of intracellular signaling pathways favoring polarization (Sosa et al., 2006; Arimura and Kaibuchi, 2007; Yi et al., 2010; Nakamuta et al., 2011; Takano et al., 2019; Rozés-Salvador et al., 2020). Most polarizing signals—if not all—are transduced by *tyrosine kinase receptors* (TRK) located at the plasma membrane, triggering the activation of the *phosphatidylinositol 3 kinase* (PI3K) and conversion of phosphatidylinositol 4,5 bi-phosphate (PIP2) into phosphatidylinositol 3,4,5 tri-phosphate (PIP3).

Polarizing neurons produce neurotrophins such as BDNF and NT3, transduced by TrkB and TrkC receptors leading to PI3K activation and further conversion of PIP2 into PIP3 (Nakamuta et al., 2011). Accordingly, neurons expressing the plasmid PH-Akt-GFP, holding the PH domain of Akt for PIP3 binding, show an enrichment of PIP3 in the axolemma (Ménager et al., 2004). Similarly, by expressing the reporter PH-Tiam1-GFP it was confirmed that both PIP3 and Tiam1 are enriched in the axon (Wang et al., 2002; Ménager et al., 2004). Remarkably, initial observations reporting these interactions were done in different models of study, including rat neurons, neutrophils and the amoebae *D. discoideum*, reinforcing the idea that the PC is conserved across species.

BDNF phosphorylates LKB1 to promote axon growth (Shelly et al., 2007), feeding the activating loop of proteins that form the PC. Similarly, the IGF-1 peptide is transduced by the IGF-1R, a TRK membrane receptor promoting the establishment of polarity (Sosa et al., 2006). Although neurons do not naturally produce IGF-1, insulin binds and activates IGF-1R, triggering the synthesis and axonal enrichment of PIP3, supporting the recruitment of Tiam1, PAR-3, PAR-6 and small GTPases in the axon (Wang et al., 2002; Nishimura et al., 2005; Sosa et al., 2006). Together, these findings support the notion that external signals converge into common pathways supporting the acquisition of polarity by producing local domains of PIP3 in the axolemma. Hence, PIP3 works as a rheostat that recruits polarity proteins. Other lipids like ceramides are also instrumental for recruiting polarity proteins such as aPKC and CDC-42 in neural precursors, although their precise role in the axonal and/or dendritic compartments remains to be tested (Wang et al., 2008). Furthermore, a detailed characterization of lipid composition in developing axon and dendrites is missing.

### Rho GTPases Bridge the Polarity Complex With MT and FA

MT and FA are usually referred to as the driving force supporting neuronal polarization (Bradke and Dotti, 1999; Conde and Cáceres, 2009; Stiess and Bradke, 2011; Schelski and Bradke, 2017). On one hand, MT assembly/stabilization and MT-based transport of Golgi-derived vesicles allow axonal extension (Conde and Cáceres, 2009; Quiroga et al., 2018), while FA confers navigation capacity to the growth cone of the nascent axon, shaping and orientating the growth in response to attractive and repulsive cues.

The reorganization of the growth cone cytoskeleton is crucial for axon formation (Bradke and Dotti, 1999; Kunda et al., 2001). Accordingly, MTs represent a platform mediating the cross-talk between the PC, small Rho GTPases and FA. An illustrative example is the case of Tiam1. In stage 2–3 neurons Tiam1 is enriched at the tip of growing axons colocalizing with PAR-3/6, but also binds to a subset of dynamic MT by interacting with MAP1B (Kunda et al., 2001; Montenegro-Venegas et al., 2010; Henríquez et al., 2012). The association with MAP1B targets Tiam1 to the tip of the nascent axon, activating Rac1 and consequently promoting axonal growth. Hence, cultured neurons isolated from MAP1B-deficient mice accumulate Tiam1 in the Soma, reducing Rac activity in axons, and restraining axonal growth (Montenegro-Venegas et al., 2010). Together, these results extend the functions of Tiam1; it not only interacts with the PC and small GTPases, but also works as a linker between MT and actin dynamics for axon development (Shi et al., 2003; Montenegro-Venegas et al., 2010). We envision a scenario where growing MT containing MAP1B and Tiam1 protrude within the central and peripheral growth cone domains of the prospective axon, leading to the interaction between Tiam1 and Rac mediated by PAR-3, PAR-6 and CDC-42 (Nishimura et al., 2005).

Interestingly, other GEFs required for neuronal polarity regulate MT dynamics. This is the case of DOCK7, a GEF selectively localized at the axon able to stimulate Rac to inactivate Stathmin, a MT-destabilizing factor, promoting axon formation (Watabe-Uchida et al., 2006). By contrast Lfc, a MT-bound GEF, activates RhoA after MT depolymerization leading to axon growth inhibition (Conde et al., 2010; Wilson et al., 2020a). Thus, MT are both signaling platforms and targets of Rho GTPases during neuronal polarization.

In addition, GTPases seem to be the convergence point of several biological process shaping neurons. For instance, physiological levels of reactive oxygen species (ROS) derived from the NADPH oxidase (NOX) complex are needed to polarize neurons and extend the axon (Wilson and González-Billault, 2015; Wilson et al., 2015, 2016, 2018). In neurons, NOX is a plasma membrane complex of 5 subunits: gp91^phox^, p47^phox^, p67^phox^, p40^phox^ and the *small GTPase Rac* (Wilson et al., 2015; Wilson and González-Billault, 2015). Accordingly, the active form of Rac allows the activation of NOX, maintaining ROS-dependent signaling needed for axon formation (Wilson et al., 2016). Although further research is needed to link both the PC and NOX complex, this example illustrates the role of small GTPases as bridges between different layers of regulation for polarity acquisition.

## The Shadows of Neuronal Polarity

Molecular mechanisms here discussed are mostly focused on protein-dependent signaling pathways. Recently, new layers of regulation have emerged controlling the acquisition of neuronal polarity, including genetics, epigenetics and biophysics. In this section, we will highlight new-old concepts usually underexplored that are instrumental to understand mechanisms shaping neurons.

### Gene Regulation: Emerging Roles of Epigenetics for Axon-Dendrite Formation

Genetic regulation shape the nervous system, but its role on the establishment of neuronal polarity has been largely unexplored (Wilson and Cáceres, 2021). Looking back the historical context in which neuronal polarization was described, parallel to the Human Project and sequencing era, the genetic regulation of polarity remained unexplored. Currently, it is known that epigenetics plays a determinant role for axonal growth by controlling the supply of gene products in time and space.

Epigenetics comprehends all mechanisms controlling gene expression by modifying the DNA, chromatin and/or transcripts without affecting DNA sequence, including DNA methylation, histone post-translational modifications and RNA stability/editing, among others. For instance, a proper balance of methylation/demethylation of histone (H) amino acid residues, such as H3K9, H3K20, H3K27 and H4K20 regulates the expression of the actin remodelers WASP and CDC-42, controlling axon extension and guidance of PVQ interneurons of *C. elegans* (Kleine-Kohlbrecher et al., 2010; Mariani et al., 2016; Riveiro et al., 2017; Abay-Nørgaard et al., 2020). Histone post-translational modifications (PMT) also impact on the polarization and axon formation of mammals in health and disease. For instance, the genetic supply of components of the RhoA-dependent signaling pathway depends on the bi-methylation of H3K9 (H3K9me2), promoting polarization (Wilson et al., 2020a). In fact, removing the H3K9me2 label blocks the transition from the multipolar to the polarized phenotype (stage 2–3) *in vitro* and *in situ*. Moreover, it leads to an overexpression of the RhoA GEF Lfc, inducing a gain of function of the RhoA/ROCK pathway; a strong inhibitor of axon formation by targeting actin dynamics (Wilson et al., 2020a). Although enzymes remodeling histones have phylogenetic differences across species, the H3K9me2 mark emerges as a common signature needed to shape axons in worms and rodents. In both species, H3K9me2 represses genes encoding actin remodelers such as CDC-42 and Lfc; all of them promoting cell and neuronal polarity.

Axonal determinants are also regulated by miRNAs (small non-coding RNAs) that control gene expression in different types of animals (Bartel, 2018). Many studies revealed that miRNAs regulate neural development and local translation of proteins in the somatodendric and axonal compartments (Cuellar et al., 2008; Dajas-Bailador et al., 2012; Iyer et al., 2014). Among several highly expressed miRNAs in the nervous system, miR-219 inhibits the translation of cell polarity regulators PAR-3, PAR-6, and aPKC (Hudish et al., 2013, 2016). In addition, miR-219 localizes differentially between the soma and the axon of neurons (Kye et al., 2007) and specifically binds to the 3′-UTR region of Tau, one of the MAPs implicated in neuronal polarization (Caceres and Kosik, 1990; Santa-Maria et al., 2015). Although miR-219 is an important regulator of neural and glial development (Zhao et al., 2010; Hudish et al., 2013, 2016; Wang et al., 2017), it is unknown whether it also regulates axo-dendritic development in neurons.

Using retinal ganglion cells of *X. laevis* it has been shown that pre-miRNAs (precursor miRNAs) are transported from the soma to the distal axon through a vesicle-based system depending on endosomal/lysosomal trafficking. This pool of pre-miRNAs is processed into mature miRNAs in response to a repulsive cue degrading the class III tubulin mRNA and reducing MT stability (Corradi et al., 2020). In line with these observations, miRNA-182 also regulates axonal guidance through a Slit2-dependent mechanism targeting the mRNA of the actin remodeler Cofilin-1, a key cytoskeleton regulator (Bellon et al., 2017). Finally, several reports showed that miRNAs also control FA and MT spatiotemporal dynamics of neuronal polarity by regulating MAP1B, Doublecortin, ROCK and GSK3β, among others (van Spronsen et al., 2013; Li et al., 2019; Lucci et al., 2020).

In addition, DNA methylation, alternative splicing and RNA editing are instrumental for shaping neurons during polarization, although downstream mechanisms are more elusive and further research is needed to unveil the precise targets that control axon-dendrite formation.

### Biophysical Factors Supporting Polarization

Currently, one of the most understudied fields controlling the polarization of neurons are the biophysical properties of the cell. For example, the anterior and posterior polarity proteins in the one-cell stage zygote of *C. elegans* are helpful to partially understand asymmetric divisions of blastocysts. However, several questions about this process still lack sound answers. For instance, it is still a matter of debate the primary signal that polarizes the zygote into the AP axis. The fusion of sperm and oocyte’s cytoplasm is one of the most relevant phenomena at this stage of development and it could be the polarizing sign defining the AP axis of the *C. elegans* one-cell zygote. Nevertheless, biophysical forces involved are just starting to be explored mostly by live-cell and super-resolution imaging techniques.

In addition to cellular and molecular factors, anisotropical forces driven by the environment might be relevant to explain the establishment and maintenance of neuronal polarity. *In vitro* axon elongation can be experimentally induced by applying tension to the tip of the growth cone trough “microelectrode towing” stretching it into the longitudinal axis at the single-cell level or integrated axon tracts (Bray, 1984; Pfister et al., 2004). Moreover, neurons show selective adhesion towards *in vitro* substrates exhibiting different stiffness, highlighting the relevance of mechanical forces during neuronal polarization (Anava et al., 2009; Ferrari et al., 2011). Studies using advanced biophysical methods have extended these observations (Nötzel et al., 2018) but still is unknown how can be integrated to understand how extrinsic physical factors determine neuronal polarity *in vivo*.

Neurons are highly compartmentalized with the cytoskeleton being a determining factor in the mechanical properties of their axons and the formation of subcellular compartments (Conde and Cáceres, 2009). Across to the axon, a periodic arrangement of actin ring-like structures separated by spectrin assemble the membrane-associated periodical skeleton (MPS) (Xu et al., 2013). These structures are also present in the AIS, which acts as a permeable diffusional barrier between the somatodendric and axonal compartments (Leterrier, 2018). In this regard, one of the most intriguing questions is what are the driving forces that spatiotemporally assemble and maintain these supramolecular structures. The intracellular milieu is a highly crowded environment and the formation of molecular condensates through liquid–liquid phase separation might be crucial in shaping and maintaining these assemblies (Hernández-Vega et al., 2017; Hayashi et al., 2021). Further studies are needed to integrate these concepts and explore how these observations could help us understand the establishment and maintenance of neuronal polarity.

### Polarization of Human Neurons

**FIGURE 2:**
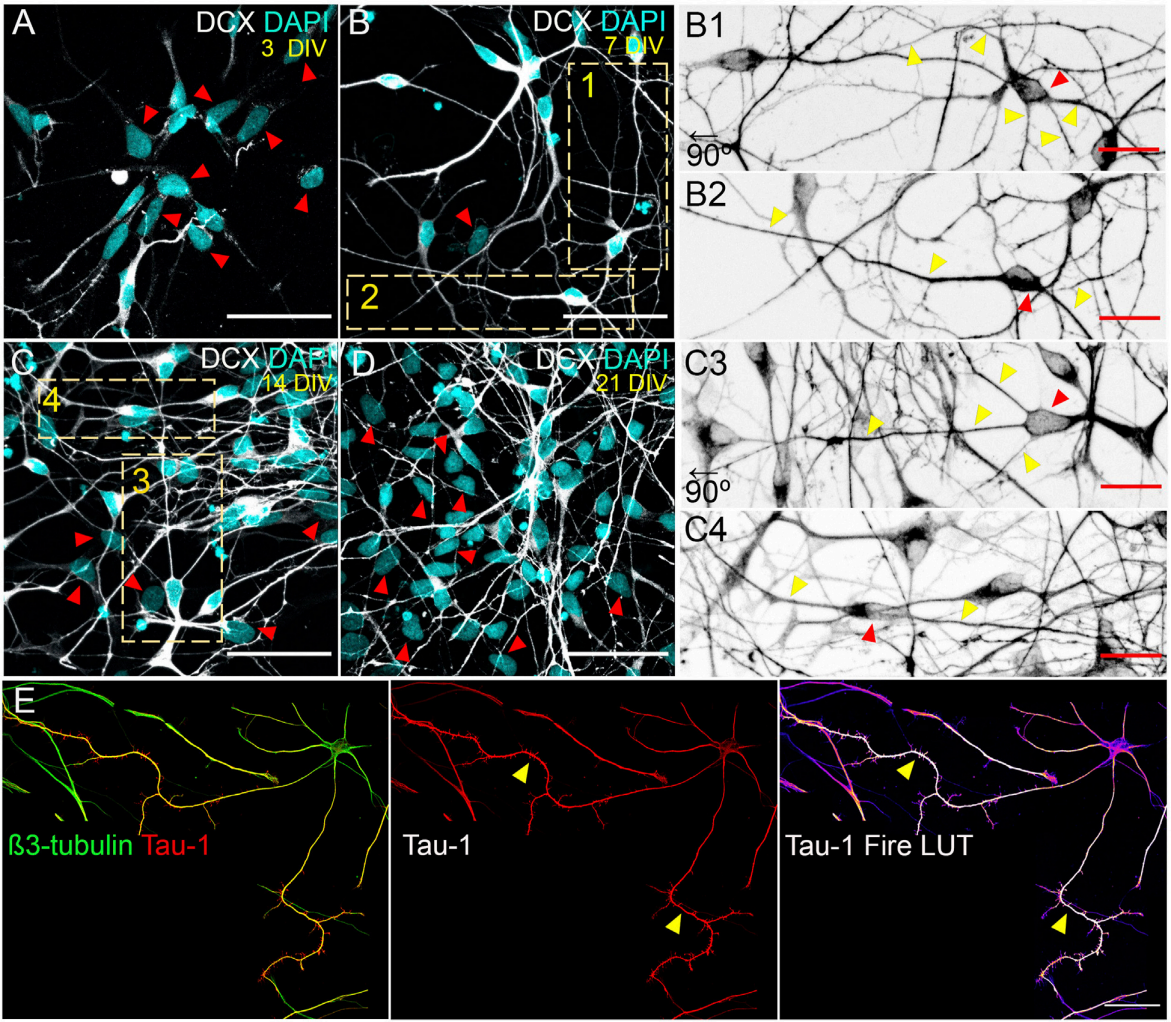
*In vitro* morphological stages of hiPSC neuronal polarization. **(A–D)** hiPSC-derived neurons at 3, 7, 14 and 21 DIV expressing Doublecortin (DCX; postmitotic neuron marker). Scale: 50 µm. Insets 1-2 (7 DIV) and 3-4 (14 DIV) are shown to visualize main morphologies detected in culture. **(B1,2, C3,4)** Magnification on insets in B and C images. Insets B1 and C3 were rotated at 90° left for aesthetic purposes. Scale: 20 µm. **(E)** Molecular polarization of neurons visualized by the axonal enrichment of the Tau-1 epitope (arrows). Representative image showing a 15 DIV hiPSC neuron exhibiting the proximo-distal gradient of the Tau-1 epitope as it occurs in rat neurons. Scale: 50 µm. hiPSCs from skin fibroblasts generated using the Yamanaka factors were gently provided by Dr. Fernando Pitossi (Fundación Instituto Leloir, FIL-CONICET, CABA, Argentina). They were differentiated into cortical neurons following established protocols (Casalia et al., 2021). Immunofluorescence and confocal microscopy was performed using established protocols (Wilson et al., 2020b).

Currently it is possible to study the polarization of human neurons using induced pluripotent stem cells (hiPSC), obtained from reprogrammed skin fibroblasts isolated from human donors (Takahashi and Yamanaka, 2006; Takahashi et al., 2007) (Figure 2). Moreover, brain organoids can be produced from hiPSC as a 3D culture system that recapitulates different cell layers that are generated during human brain development (Lancaster et al., 2013). Although early works with human neuroblastoma cells have been instrumental to characterize biochemical mechanisms, morphological and cellular properties are notoriously different compared to primary neurons isolated from animal tissues. Moreover, some biological mechanisms are not broadly conserved among species and significant differences might arise through studies based on hiPSC-derived neurons (Rolls and Jegla, 2015; Kshirsagar et al., 2019). Therefore, these novel models will allow us to assess whether conserved mechanisms discussed here also shape the architecture of human neurons in health and disease.

hiPSC-derived neurons and brain organoids are being extensively used in disease modeling since they could recapitulate many aspects of human diseases (Shi et al., 2017; Penney et al., 2020). The ability to generate any CNS cell type *in vitro* from hiPSC is particularly relevant in neurobiology due to limited access to primary cells from human CNS. Moreover, the implementation of 3D culture brain organoids from patient-derived hiPSC will unravel the complexity and pathophysiological relevance of cell–cell interactions in brain diseases. A broad range of applications have been already assessed including drug discovery and development, toxicology and virology (Rivetti di Val Cervo et al., 2021). However, and with some exceptions, only a few studies focus on central questions about the biology of neural development including the establishment and maintenance of neuronal polarity.

Two recent reports examine how hiPSC neurons polarize with similarities and differences compared to murine neurons *in vitro* (Lindhout et al., 2020, 2021). For example, the timing of polarity acquisition is significantly slower. Whilst rat cultured neurons take approximately 36 h in defining the axonal compartment, human cultured neurons require several days (Figure 2A). In this regard, rat and hiPSC neurons show differences. For instance, the rat culture begins with postmitotic neurons isolated from E18.5 hippocampi. In contrast, hiPSC neurons are obtained from NSC differentiation *in vitro*; consequently, NSC need at least a couple of days to turn off the stem phenotype to generate neurons. In addition, the culture of hiPSC neurons is heterogenous; although mainly enriched in neurons, it also contains non-neuronal cells even after several weeks (Figures 2A–D, red arrows). By contrast, the culture of rat neurons is highly pure (over 95% of cells are neurons).

Despite differences mentioned above, hiPSC and rat cultured neurons share common morphologies. For example, in both models, neurons develop a pyramidal phenotype (Figures 2B1,C3) showing the classical distal enrichment of Tau-1 in the axon (Figure 2E, arrows). In addition, bipolar morphologies are also detected (Figures 2B,C4). Thus, our first approaches culturing hiPSC neurons suggest differences and similarities that need consideration. Significantly, culture and differentiation protocols will require standardization across laboratories to successfully apply hiPSC-based models for future studies uncovering the fundamentals of neurobiology in health and disease.

## Main Conclusion and Perspectives

In this article we attempted to do a critical review on the most fundamental mechanisms supporting the polarization of neurons. Undoubtedly, the PC exerts a deep influence on shaping neurons as well as on polarizing cells and tissues of all animals. Phylogenetic preservation of *Par* genes reinforces their importance as a genetically-encoded system to develop asymmetries as a mechanism to gain functional specialization throughout development beyond species and evolution. Cell-context dependent mechanisms will determine the outcome of polarization and there are intrinsic and extrinsic elements switching on/off the PC. In neurons, this circuitry is modulated by external signals such as neurotrophic factors, TrK receptors at the membrane and cell-cell interactions, among others. Intrinsically, the repertoire of neuron-specific proteins should also be carefully analyzed. Unfortunately, a detailed proteomic analysis of polarizing neurons is missing, which would be instrumental to understand the orchestration of protein networks during development. For example, DRG neurons express a specific splicing variant of Tau protein [named high-molecular weight (HMW) Tau], which includes the exon 6 (skipped in central neurons) (Mavilia et al., 1993). However, the role of this variant on MT stability, morphology and function remains unknown. Similarly, transcriptomic-based studies have been also elusive, although recent works are considering these approaches. Thus, the transcriptomic and proteomic landscapes should be considered to fully understand mechanisms supporting neuronal polarity.

After revisiting seminal papers we noticed that cell biology is not enough to explain (by itself) the polarization of cells and tissues, including neurons. In other words, protein-protein interactions and intracellular signaling solve a part of a complex problem. Novel hypotheses are beginning to include the genetic regulation and nuclear events into the polarity equation, filling gaps and enriching previous mechanisms. In addition, biophysics—unfortunately one of the most underexplored dimensions of neuronal polarity—will be critical to understand the contribution of the cytoplasm and membranes in shaping neurons. Moreover, culturing human neurons is an opportunity to evaluate the contribution of ancient molecular systems such as the PC, which would reinforce their evolutionary relevance in shaping cellular morphologies independent of species. Thus, mixing old and new hypotheses, emerging and classical experimental models and involving complementary disciplines will be needed to unveil secrets supporting the polarization of neuronal and non-neuronal cells.
